# A comparison of temporal pathways to self-harm in young people compared to adults: A pilot test of the Card Sort Task for Self-harm online using Indicator Wave Analysis

**DOI:** 10.3389/fpsyt.2022.938003

**Published:** 2023-01-12

**Authors:** Joanna Lockwood, Camilla Babbage, Katherine Bird, Imogen Thynne, Andrey Barsky, David D. Clarke, Ellen Townsend

**Affiliations:** ^1^National Institute of Health Research MindTech MedTech Co-operative, School of Medicine, University of Nottingham, Nottingham, United Kingdom; ^2^School of Psychology, University of Nottingham, Nottingham, United Kingdom; ^3^School of Computational Biology, University of Birmingham, Birmingham, United Kingdom

**Keywords:** self-harm, adolescence, adulthood, card sort, Indicator Wave Analysis, CaTS-online, digital interventions, co-development

## Abstract

**Background:**

Self-harm is complex, multifaceted, and dynamic, typically starts in adolescence, and is prevalent in young people. A novel research tool (the Card Sort Task for Self-harm; CaTS) offers a systematic approach to understanding this complexity by charting the dynamic interplay between multidimensional factors in the build-up to self-harm. Sequential analysis of CaTS has revealed differences in key factors between the first and the most recent episode of self-harm in adolescence. Rates of self-harm typically decline post-adolescence, but self-harm can continue into adulthood. A comparison between factors linked to self-harm in young people vs. adults will inform an understanding of how risk unfolds over time and clarify age-specific points for intervention. A pilot online adaptation (CaTS-online) and a new method (Indicator Wave Analysis; IWA) were used to assess key factors in the build-up to self-harm.

**Methods:**

Community-based young people (*n* = 66; 18–25 years, *M* = 21.4; *SD* = 1.8) and adults (*n* = 43; 26–57 years, *M* = 35; *SD* = 8.8) completed CaTS-online, documenting thoughts, feelings, events, and behaviours over a 6-month timeline for the first ever and most recent self-harm. A notable interdependence between factors and time points was identified using IWA.

**Results:**

Positive emotion at and immediately after self-harm exceeded the threshold for both groups for both episodes. Feeling better following self-harm was more pronounced for the first-ever episodes. Impulsivity was an important immediate antecedent to self-harm for both groups at both episodes but most markedly for young people. Acquired capability was notable for adults’ most recent episodes, suggesting this develops over time. Burdensomeness was only more notable for adults and occurred 1 week prior to a recent episode. Both groups revealed patterns of accessing support that were helpful and unhelpful.

**Conclusion:**

Commonalities and differences in the temporal organisation of factors leading to and following self-harm were identified in young people and adult pathways which shed light on age-specific factors and possible points of intervention. This has implications for clinical support and services around approaches to positive feelings after self-harm (especially for first-ever self-harm), feeling of burdensomeness, impulsivity, and acquired capability leading up to self-harm. Support is provided for card-sort approaches that enable the investigation of the complex and dynamic nature of pathways to self-harm.

## Introduction

### Self-harm across adolescence and adulthood

Self-harm (non-fatal intentional self-injury or self-poisoning regardless of the motivation or intent associated with the act) (1) is a complex and common behaviour in adolescence (the developmental stage from around 11–25 years of age) which is recognised to correspond to the social, psychological, neurodevelopmental, and biological growth undertaken between childhood and adulthood (2). Around one in five youth report having self-harmed at least once (3), although this figure is likely to underestimate true prevalence given that for many young people self-harm remains hidden at a community level (4). Self-harm behaviour typically starts and peaks during early- (11–14 years) to mid-adolescence (15–18 years) and is associated with risk of repetition, potential life-long adverse outcomes, future mental health conditions, and, substantially increased likelihood of suicidal thoughts and behaviour (3, 5–9). Rates of self-harm are also higher among adolescent than child or adult populations, particularly in mid and late adolescent groups, and have risen sharply in recent years (10, 11). Population-based cohort studies charting the natural history of self-harm clearly indicate that the frequency of self-harm substantially decreases during the transition from late adolescence to early adulthood (9, 12–14). The prevalence, lifelong consequences, and the risk to life in youth underscore the importance of a research focus on clarifying risk and protective factors for self-harm salient during this key transitional phase.

Yet, while less common in adulthood, self-harm is nonetheless present and also on the rise in adult groups (15, 16). In fact, rates and risk factors for self-harm across adult populations, particularly, in older adulthood remain under-researched, especially within community-based studies. There may be distinctions in risk profiles that underlie adult- vs. adolescent-presenting self-harm. Self-harm in older adults compared to adolescents certainly appears associated with higher lethality, conferring a 67 times greater risk of completed suicide (17). Diminishing self-harm in adulthood may also relate to the substitution of self-harm with other risky behaviours, such as problematic drinking (13). Arguably, self-harm, which continues into adulthood or adult-incident self-harm, may be characterised by distinct emotional, behavioural, or environmental self-harm pathways from adolescent-only self-harm, which suggest opportunities for targeted intervention and treatment.

### Risk factors across the life-span

A substantial body of empirical and theoretical work supports an increased understanding of the influencing factors associated with self-harm and suggests self-harm results from a complex interplay between various genetic, biological, psychological, psychiatric, cultural, and sociodemographic factors that act in concert to confer risks (18) and which are developmentally charged and fluid. Psychiatric disorders, particularly mood and anxiety disorders, and psychological factors (including low problem-solving, low self-esteem, impulsivity, vulnerability to hopelessness, and a sense of entrapment) are recognised as contributing to vulnerability to self-harm across the lifespan (19–22). Psychological vulnerability for self-harm in young people is proposed to relate to developmental changes in childhood and adolescence, which undermine emotional control and coping with stress (12), with pubertal stage—rather than age—the critical contributory factor (23). By adulthood protective factors such as affective stability, emotion regulation and increased behavioural inhibition may typically temper this vulnerability (24). Psychological vulnerability for self-harm in older adults is characterised by feelings of burdensomeness, isolation, loneliness, hopelessness and loss of control and the impact of factors (physical/social/economic) preceding self-harm which relate to older life-stage challenges (17, 25, 26). Consistent evidence has clarified that self-harm often occurs in the context of one or multiple negative life events that are typically social/interpersonal and which also differ by age, such as family and peer group problems in children and younger adolescents, or spouse/partner problems in older adolescence and adulthood (5).

Longitudinal cohort studies have advanced understanding of the developmental history of self-harm and improved the identification of biopsychosocial causes for self-harm over time, charting distinct behavioural and emotional risk profiles. Moran et al. revealed that although most self-harm spontaneously resolves by adulthood, persistence into adulthood is associated with high anxiety and depressive symptomatology in adolescence (12). Research that continues to elucidate how characteristics of self-harm unfold over time may help to pinpoint salient intervention points.

### Accounting for complexity and temporal dynamics

Despite considerable research focus on potential factors influencing self-harm, the accurate prediction of who is likely to self-harm and when they are likely to do so remains poor (27). In part, this may be due to a methodological over-reliance on simple cross-sectional associative studies and examination of factors associated with self-harm in isolation, which limits their predictive utility (27, 28). Such approaches fail to account for the complexity of risk factors or the dynamic temporal context in which these factors are likely to confer their risk. In response, recent strides in novel research approaches are improving our ability to understand, predict, and prevent self-harm, including those employing real-time monitoring methods which follow the dynamic course of self-harm within a natural environment at an individual level (29).

The Card Sort Task for Self-harm (CaTS) (30) is a research tool that takes a dynamic approach to map the influence of multiple potential distal and proximal biopsychosocial factors that precede an episode of self-harm. CaTs offers a way of examining patterns of thoughts, feelings, events, and behaviours experienced as salient at an individual level through the positioning of cards along a timeline, providing a nuanced understanding of how and when the risk for self-harm emerges and progresses. The approach reflects important contemporary explanatory models that account for the multidimensional nature of self-harm and the transition from vulnerability, to thinking about self-harm (ideation) and to acting on those thoughts (behavioural enaction) (31–34). In a first test of the CaTS, Townsend et al. used a lag sequence analysis statistical technique to identify the important sequences of items leading to a first-ever and the most recent episode of self-harm in a sample of 45 young people. This distinction in episodes allows reflection on the transition from ideation to behavioural enaction for the first onset of self-harm and highlights maintained factors of risk over time. Sequence statistical approaches, such as lag sequence analysis, analyse the transitions between one event (antecedent) and the following event (the sequitur) and enabled Townsend and colleagues to show that factors most proximal to self-harm in young people were negative emotions, impulsivity, and having access to means. They also identified important distinctions in significant sequential structures between the first-ever and most recent self-harm. For example, hopelessness was an important antecedent of behaviour in the most recent episode of self-harm, but while the first-ever self-harm was associated with feeling better after self-harm, this was no longer the case by the most recent episode. Additional examination of temporal dynamics in self-harm using the CaTS tool within research settings could extend the examination of pathways to self-harm in adolescents *versus* adult groups and offer a method of targeting developmentally specific points for intervention in the self-harm pathway.

### The current study

This study uses an internet-mediated version of the CaTS (CaTS-online) to systematically compare the dynamic interplay of factors that lead to self-harm in young people (18–25 years) and adults using a longitudinal design. Traditionally, CaTS is a manual task with cards presented along a timeline in a tabletop manner. An online adaptation of CaTs could offer increased functionality and scope, including a more efficient process of recording, coding, and tracking the order and frequency of cards, and the capacity to allow for multiple uses of the same card at different time points, which was not a feature of the manual version. Previous tests of the CaTS have focused on relatively small, clinical, or targeted populations. An online version extends the capacity to access a wider, more diverse participant pool (35) and allows the task to be performed anonymously and in less-formal settings (36). Given the remote nature of CaTS-online, the study will draw on adult and late adolescent (18+) groups, herein specified as “young people.”

Novel approaches to analyse the multi-dimensional nature of risk over time are necessary to advance the understanding of when, why, and who is at risk of self-harm. A limitation of the sequence analysis approach previously employed by Townsend et al. (30) was that the use of the same card at multiple time points was prohibited, a restriction noted by young people in anecdotal feedback and in Patient Public Involvement work used to develop the original CaTS (30). To address this issue, the present study uses Indicator Wave Analysis (IWA) as a method of temporal measurement, which allows multiple, simultaneous, and sequential events to be analysed across varying time spans (37). In addition, IWA produces easy-to-interpret wave diagrams (known as indicator wave diagrams) that provide a profile of the factors (indicators) absent or prevalent relative to other indicators at a time point (37). The use of IWA in psychological methods is novel, but as a method of allowing complex data to be plotted and examined in simplified diagrams, it is an attractive approach to aid the interpretation and discourse of the fluctuating and complex nature of self-harm and suited to analyse the CaTS-online data. As IWA is a novel approach, and there is limited data specifically comparing adults and young people who self-harm, no specific predictions are made concerning the absence or presence of items across time. An additional aim of this study was to explore what can we learn from CaTS-online as a research tool and the potential for application of IWA as an analysis technique to support further development of CaTS.

### Research questions

1. Is there an association between cards that are selected as part of the CaTS and the timepoint leading up to and after self-harm episodes in adults and young people?
2. Do items at time points leading up to and after self-harm differ between adults and young people?
3. Do items at time points leading up to and after self-harm differ between the first ever and the most recent episode of self-harm for adults and young people?

## Materials and methods

### Recruitment

Individuals with lived self-harm experience and access to the internet, and aged 18 years or more were invited to participate in the study *via* social media advertisements through gatekeepers (national self-harm support organisations). Undergraduate students at the University of Nottingham were also invited to take part in return for course credits. Participants received an online information sheet and completed the online consent form and were then invited to complete CaTS online. This research adhered to and was approved by the University of Nottingham, School of Psychology Research Ethics Committee (Ref: F967).

### Adaptation of CaTS

The CaTS is a card sort task of 117 cards grouped broadly into sub-sets describing thoughts, feelings, events, behaviours, services and support, and items relevant to and after self-harm. Groupings allow participants to easily navigate the cards provided. CaTS was developed in conjunction with academic and clinical experts and an advisory group of young people with lived experience, and it draws on insight from key theoretical models and empirical evidence. During the task, the participant is asked to select and organise cards that are salient to a specific self-harm episode chronologically along a 6-month sequential timeline. Timestamps are provided (*6 months before, 1 month before, 1 week before, 1 day before, 1 h before self-harm, and afterwards*) [Refer Ref. (30) for more detailed information about CaTS].

**FIGURE 1:**
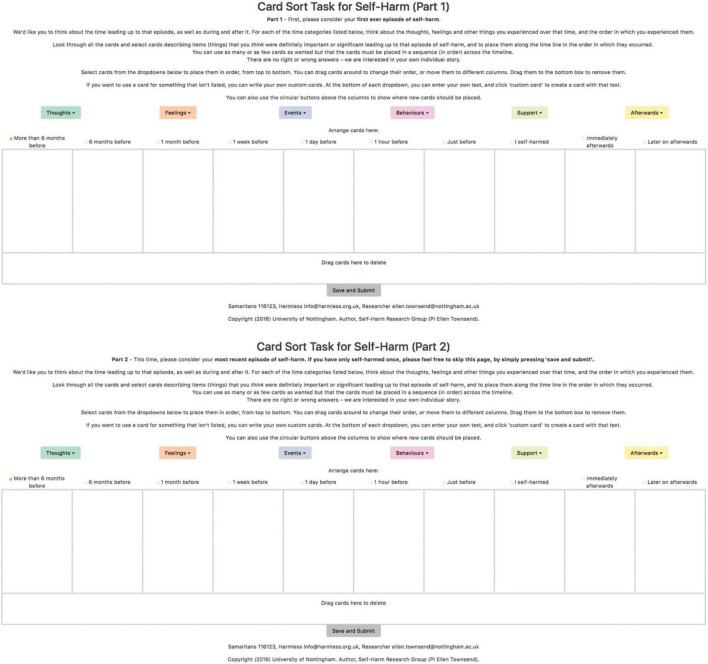
A screenshot of the first ever and most recent episode of self-harm tasks for CaTS-online.

The CaTS-online was intended to replicate the manual version of CaTS with a modification to the format which extended the timeline to run *over 6 months before, 6 months before, 1 month before, 1 week before, 1 day before, 1 h before, just before self-harm, immediately afterwards*, and *a later on after.* This modified timeline was suggested by a PPI focus group with young people that explored ways to develop the original CaTS. The timeline was presented graphically as a table with timestamps as the heading of each column, as shown in Figure 1. Each sub-set was presented above the timeline screen. When clicked, items in the sub-set would appear in a drop-down menu in random order. The participant could then drag and drop cards from each sub-set into the relevant timeline column. Participants were able to delete cards that were placed incorrectly. As with the manual version, participants were able to use as many or as few cards as they wanted and were able to create their own cards by selecting “custom card” at the bottom of the drop-down list. Uniquely, CaTS-online allowed participants to use the same card multiple times. On completion of the task, a custom string of data including the time point and cards selected during the task was created. These data were only accessible to the research team.

As CaTS-online is completed remotely, signposting information with contact details for the available support organisations (including those acting as gatekeepers to the study) was included at the bottom of each of the CaTS-online windows, along with contact details for the Principal Investigator (ET). An initial prototype of CaTS-online, which was created and piloted with Undergraduate students at the University of Nottingham and members of the research team (ET, IT) and PPI group necessitated no format or delivery changes.

### Study procedure

At the start of the study, participants were asked for their demographic information (age, date of birth, sexuality, gender, ethnicity and country of residence, and current employment/education status). Closed response questions asked participants to estimate how many times they had self-harmed across 9 options increasing incrementally from once to more than two hundred and if they had used “self-injury,” “self-poisoning,” or “other” methods of self-harm [adapted from Wadman et al. (38)]. An open-response box could be used to record other methods of self-harm. Open-response boxes were also used to measure participant estimations of how long ago they had first and most recently self-harmed in years, months, and days. Data were collected between December 2017 and February 2018. All data, including consent, and completion of the card-sort task was captured by the CaTS-online programme in the same session.

Participants were asked to complete the CaTS-online task twice, reflecting on their first-ever and most recent episode of self-harm in line with previous studies, as shown in Figure 1 (30, 38).

In order to monitor the impact of taking part in the study, participants were required to rate their current emotional state at the start and end of the study by completing a Visual Analogue Scale (VAS) presented as a scale ranging from 0 (illustrated with an unhappy face) to 10 (illustrated with a smiling face). Participants were provided with the prompt: “How are you feeling right now?.” They were asked to reflect on their VAS rating and any change in their mood and were provided with signposting information and prompts for self-care. Cute animal pictures were also included at the end of the task as part of the final debrief in line with recommended practice to support mood mitigation in sensitive research (39).

### Data analysis

#### Data collation and pre-statistical manipulation

Data were downloaded as a.CSV file and imported into a spreadsheet programme. Participants were divided into two age categories, young people (18–25 years) and adults (26 years and over). Data were organised into separate time points across the timeline, for the most recent and first-ever self-harm episode.

Individual analysis of each card would be too complex for IWA, given the high volume of cards in the CaTS (*n* = 117) (37). Therefore, the complexity of data analysis and figures was reduced [as in previous sequence-based approaches (30, 38)] by grouping items into categories. This process was initially completed by two Psychology Undergraduates, who were part of the research team, and then reviewed further with the wider research team, leading to 17 categories being developed such as “negative life events or social problems” and “exposure” (refer to Supplementary material 2 for details of which cards were placed into which categories). A total of 89 (76%) of the 117 cards were categorised. In the interests of parsimony, cards were prioritised, which fitted conceptually into categories or were retained where they were considered of theoretical relevance for the current study, as agreed by consensus in the research team.

#### Indicator Wave Analysis

Indicator Wave Analysis (IWA) can be used to assess whether an event is interdependent on another by analysing multiple behaviours and events along a time scale (37).

**FIGURE 2:**
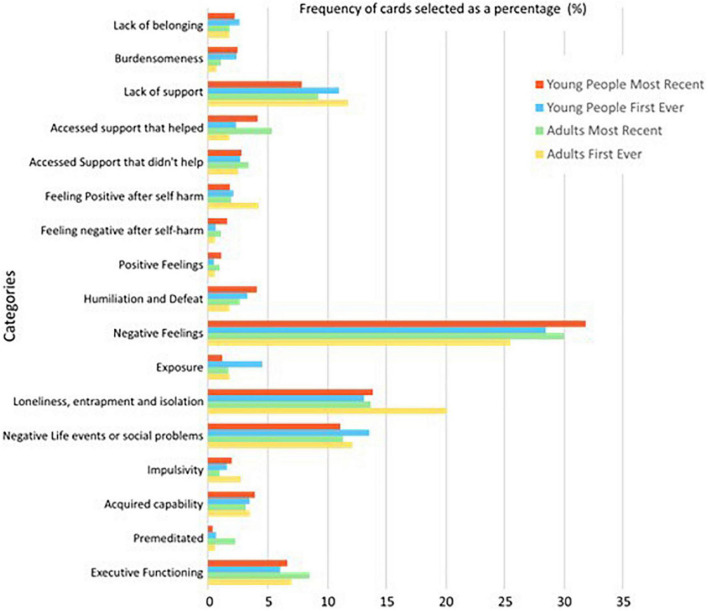
A graph displaying the frequency of cards, pooled as a percentage of cards in each category, across each of the four groups.

The process of IWA begins with “unitisation” to divide items into related categories. Second, items were organised into timepoints, e.g., “1 h before,” along the timeline. Frequencies were then calculated for items in each time point, leading up to the action/behaviour. For each card, the frequency of that card occurring at a particular time point was calculated for both age groups during their most recent and first-ever episode of self-harm. A frequency table of the number of occurrences of cards within a category across the timeline was also produced. For each of the four groups, percentages were calculated based on the total number of cards within that category to produce comparable graphs due to differences in the number of cards selected between the four conditions (Figure 2).

Finally, to produce standardised residuals (SRs), a chi-square analysis was conducted in SPSS (2021, Version 28.0) for young persons’ and adults’ most recent and first-ever self-harm episodes. SRs are used as descriptive variables giving a measure of the strength of the difference between observed and expected values to highlight points of interest by indicating when an event is happening more or less frequently than would be expected by chance. Requirements for the chi-square are not presented here as the outcome of the chi-square statistic is not required for this method.

These identified which categories were disproportionately more or less likely to occur than might be expected by chance at specific time points, suggesting an association of cards within a category being selected at a time point more frequently than would be picked up by chance. A threshold value of ≥2.0 was used to identify an association, in line with the convention for the evaluation of categorical data using chi-square (40). All data were input by CB and checked by KB for inconsistencies.

### Incomplete data

In total, 101 participants completed the CaTS for their first-ever self-harm episode, and 96 participants completed the CaTS for their most recent self-harm episode. This sample size is consistent with previously published research utilising CaTS and sequence analysis techniques (30, 38). There was a low level of missing data; four out of 66 young people and four out of 43 adults did not complete the CaTS for their first-ever episode of self-harm. A total of 11 young people and two adults did not complete the CaTS for their most recent self-harm episode. Analysis proceeded on the remaining completed CaTS data.

## Results

### Participant characteristics

A total of 109 people participated, aged 18–57 years (*M* = 26.8 years; *SD* = 8.7). In the young people group (*N* = 66; Age: *M* = 21.4 years; *SD* = 1.8), there were 58 women (87.9%), 4 men (6.1%), 3 transgender individuals not identifying as either male or female (4.5%), and 1 transgender male (1.5%). The adult group (*N* = 43; Age: *M* = 35 years; *SD* = 8.8) included 30 women (69.8%), 10 men (23.2%), 2 transgender individuals not identifying as male or female (4.7%), and 1 participant who was unsure (2.3%).

Across both groups, participants self-reported as “heterosexual” (56.9%), “bisexual” (23.9%), and “homosexual” (10.1%). Participants reported their ethnicity as “White British” (79.8%) or “Other White” (11.9%). A total of 51.4% were in full time education, 38.5% were employed, and 10.1% were not employed.

### Method, frequency, and onset of self-harm

The age at the first self-harm episode ranged between 7 and 21 years (*M* = 14.6 years; *SD* = 3.1) for young people and between 7 and 46 years (*M* = 17.5 years; *SD* = 8.4) in the adult group. The average duration was 5 years 6 months for young people (range 1 month–13 years, 4 months), and 15 years 7 months (range 1 month–48 years) for adults. Almost all participants across the total sample had their first self-harm episode in adolescence, with only 4/43 (9%) reporting adult incident self-harm. Self-injury was the most common method of self-harm, reported by all participants. Additionally, 16 young people (24.2%) and 14 adults (32.6%) reported using self-poisoning (other methods of self-harm, reported by 13 participants completing the CaTS, are detailed in Supplementary material 3). Out of 66, 33 young people (47%) and 30 out of 43 adults (70%) reported self-harm in the last 6 months. All participants reported repeat self-harm except 2 adolescents who reported only one episode of self-harm, and 2 participants who did not provide a response.

### Frequency analysis

In total, young people selected 1,977 and 1,233 cards, and adults selected 818 and 814 cards in relation to their first-ever and most recent self-harm, respectively. The frequency percentage of cards selected in each category for each group is displayed in Figure 2. The category “Negative Emotion” accounted for the largest percentage of cards selected across all groups, with a peak of 31.96% of total cards selected by young people distributed within this category for most recent self-harm.

Table 1 presents the top 10 most frequently selected cards for both groups at both time points. Notably, for the first and most recent episode of self-harm, feelings of depression and sadness were frequently reported by both adults and young people, and self-hatred was also a prominent shared selection, markedly for young people. Both groups also reported not being able to tell anyone how they were feeling, and this was highly selected for the first-ever and most recent self-harm. “I felt exhausted” and “I was angry” were often selected by both adults and young people in relation to the most recent, but not the first-ever, self-harm. Other highly occurring card selections for both groups included wanting to die, feeling anxious, and feeling worthless.

**TABLE 1:** A table displaying the most frequently selected cards, across the four groups.

	Position	Card #	Card description	Frequency
**Adult first ever**	1	B04	I felt depressed and sad	53
	2	A09	I could not tell anyone how I was feeling	48
	3	B10	I hated myself	47
	4	B20	I felt like I did not belong	36
	5	B14	I felt trapped	35
	6	F06	I felt better after self-harm	35
	7	D12	I isolated myself from others	34
	8	A01*	I wanted to die	32
	9	A03	There was no one to turn to for help	32
	10	A04	I could not trust anyone	29
**Adult most recent**	1	B10	I hated myself	54
	2	B04	I felt depressed and sad	42
	3	B05	I felt very anxious	42
	4	B03*	The mental pain was unbearable	39
	5	A09	I could not tell anyone how I was feeling	38
	6	D12	I isolated myself from others	37
	7	B08*	I felt exhausted	36
	8	B01	I was angry	34
	9	B06	I felt worthless	32
	10	A03	There was no one to turn to for help	30
**Young people first ever**	1	B04	I felt depressed and sad	132
	2	B10	I hated myself	104
	3	B06	I felt worthless	96
	4	A03*	There was no one to turn to for help	89
	5	A09	I could not tell anyone how I was feeling	84
	6	B05	I felt very anxious	81
	7	A01*	I wanted to die	77
	8	B03	The mental pain was unbearable	71
	9	B25*	I felt numb	60
	10	B07	I felt disgusting	57
**Young people most recent**	1	B05	I felt very anxious	61
	2	B08*	I felt exhausted	47
	3	B04	I felt depressed and sad	99
	4	B06	I felt worthless	59
	5	A09	I could not tell anyone how I was feeling	50
	6	B10	I hated myself	73
	7	B01	I was angry	45
	8	B16	I felt I could not escape from feelings or situations	49
	9	A01*	I wanted to die	67
	10	B18*	I felt very hopeless about the future	44

*Indicates cards that were not categorised.

Frequencies for all cards, including those that were not chosen by participants across either self-harm episode, can be seen in Supplementary material 4–Frequency of Individual Cards. Examination of item frequencies outside of those most frequently endorsed is also noteworthy in highlighting the differential relevance of individual items for age groups and self-harm episodes where categories include multiple items. For example, frequency data suggest that mental abuse and bullying within the category “Negative life events and social problems” are more prominent items for the first-ever self-harm in young people than for recent self-harm. Relatively, low-level endorsement of cards among participants is also of importance. For example, in general, endorsement of items relating to support, irrespective of whether it was helpful or not, was low, indicating modest levels of support seeking broadly and, in particular, little endorsement of support through formal (clinical/education) settings.

### Indicator Wave Analysis graphs

Standardised residuals, obtained from the chi-squared statistic, are used to develop a (category × timepoint) frequency matrix and to enable the development of IWA graphs as a guide to points of interest rather than to report an association between variables. Nonetheless, the chi-squared statistic is presented in the following text (Table 2), detailing the significant association between categories and time for each group.

**TABLE 2:** The chi-squared statistic of associations between categories and time points for each group with violations reported.

Condition	Chi-squared value (to one d.p.)	Degrees of freedom	Significance level
Young people, ≤25 years (Most recent)	815.8^a^	144	*p* < 0.001
Young people ≤ 25 years (First ever)	1202.5^b^	144	*p* < 0.001
Adults, ≥26 years (Most recent)	652.2^c^	144	*p* < 0.001
Adults, ≥26 years (First ever)	738.9^d^	144	*p* < 0.001

^a^104 cells (61.2%) have an expected count of less than 5. The minimum expected count is 0.32.

^b^73 cells (42.9%) have an expected count of less than 5. The minimum expected count is 0.51.

^c^119 cells (70.0%) have an expected count of less than 5. The minimum expected count is 0.56.

^d^122 cells (71.8%) have an expected count of less than 5. The minimum expected count is 0.33.

**FIGURE 3:**
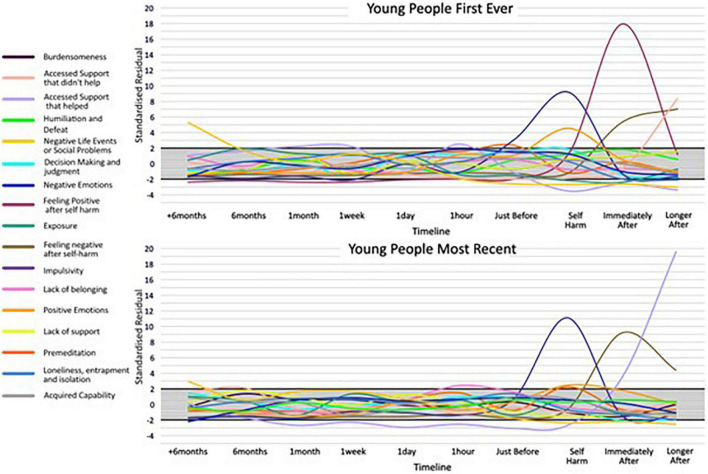
Indicator Wave graphs showing the temporal organization of categories where at least one time point reached ± 2 for young people first ever and most recent episode of self-harm, SRs ± 2 indicating higher or lower frequency than would be expected by chance.

**FIGURE 4:**
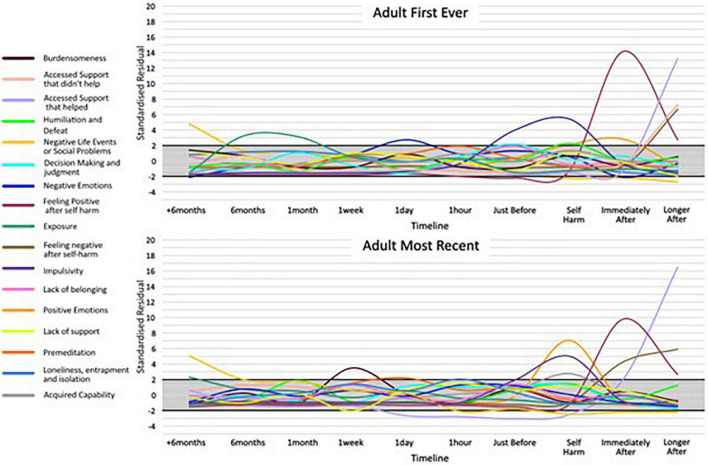
Indicator Wave graphs showing the temporal organization of categories where at least one time point reached ± 2 for adult first ever and most recent episode of self-harm, SRs ± 2 indicating higher or lower frequency than would be expected by chance.

**FIGURE 5:**
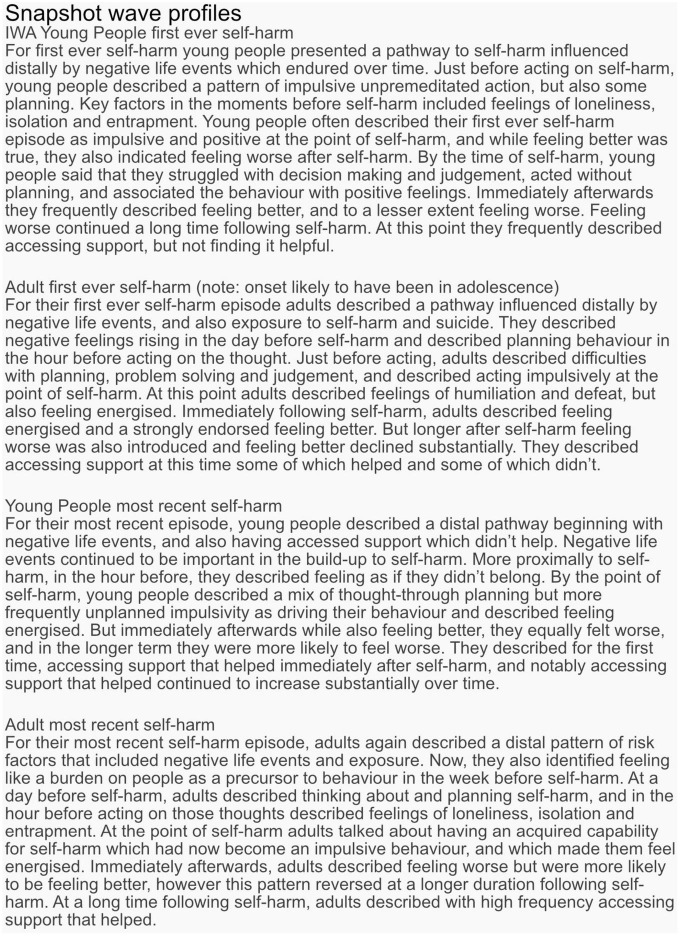
Snapshot wave profiles based on notable category selection by time point.

Across the 17 categories, 13 categories had SRs that indicated cards in the card category were selected more frequently than would be expected by chance, at particular time points along the timeline for an adult’s first-ever episode of self-harm. Similarly, 12, 11, and 12 card categories reached the notable threshold for adults’ most recent, young people’s first ever, and young people’s most recent episodes of self-harm, respectively. This suggests an association between cards relating to particular thoughts, feelings, events, and behaviours leading up to and after self-harm, across adults and young people. For the categories where an SR reached a higher or lower than expected frequency, an IWA graph is presented (Figures 3, 4) and these are narratively described in a snapshot wave profile in Figure 5. An Indicator Wave frequency matrix showing the SRs of categories occurring more or fewer times than expected by chance across timestamps is included in Supplementary material 5. IWA graphs summarising all categories, i.e., including those where SRs did not exceed the threshold, are included in Supplementary material 6.

**FIGURE 6:**
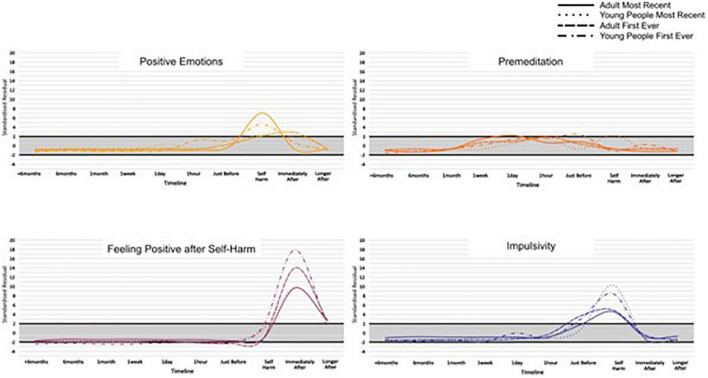
Indicator Wave graphs comparing standardised residuals (SRs) for each group across the timeline, for the categories: Positive emotions, premeditation, feeling positive after self-harm, and impulsivity.

**FIGURE 7:**
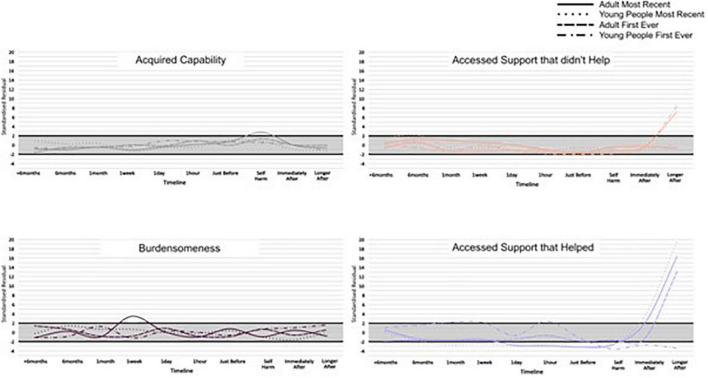
Indicator Wave graphs comparing the standardised residuals (SRs) for each group across the timeline, for the categories: Acquired capability, burdensomeness, accessed support that didn’t help, and accessed support that helped.

For key categories of interest, Indicator Wave graphs are presented (Figures 6, 7) displaying the SRs for young people and adults at each time point for the category. These graphs visually highlight key indicators of relative importance at time points and allow for comparison across groups. For example, the card category “accessed support that didn’t help” exceeded the threshold, occurring more often than might be expected by chance relative to other indicators, immediately after self-harm, and this was the case for both groups for the first-ever but neither group for the most recent episode of self-harm. For adults, there is a notable spike reaching above the threshold at the “accessed support that helped category” on both timelines (Indicator Wave graphs for all remaining categories are presented in Supplementary material 7).

### Visual Analogue Scale

The mean score on the emotional state VAS before completing the CaTS for young people was 5.32 (*SD* = 2.13) and 4.79 (*SD* = 1.97) after completion. The mean emotional state score for adults was 4.93 (*SD* = 2.79) before completion and 4.50 (S*D* = 2.79) after completion. Thus, scores persisted around the mid-range of the scale for both groups before and after completion. For young people, this represented a significant decrease in mood [*t*(56) = 2.49, *p* = 0.02], but no significant change in VAS scores was observed for adults [*t*(39) = 1.50, *p* = 0.14].

## Discussion

This exploratory study reports early findings using a novel analysis approach (Indicator Wave Analysis) to chart and aid understanding of the temporal dynamics of biopsychosocial factors associated with self-harm. Using the prototype CaTS-online, we were able to describe the salient factors building up to and following self-harm for young people and adults’ first-ever and most recent self-harm pathways. Our findings indicate that there is an association between factors leading up to self-harm episodes in adults and young people at specific time points and suggests similarities and differences between these groups that unfold over time. This replicates and extends earlier work demonstrating the sequential pattern of self-harm using CaTS (30).

While raw frequencies support an understanding of which multiple factors are consistently identified as associates of self-harm, the IWA analysis allows an examination of their temporal importance, relative to other indicators, in the months, weeks, days, hours, and moments preceding and after self-harm. In terms of negative and positive emotions, the Indicator Wave diagrams provide a clear indication of the temporal distribution of relevance to established affect regulation models of self-harm (41, 42). Positive emotions (feeling energised) first reach the positive threshold value from just before and at the point of self-harm and continue to rise before diminishing in value. Of note, for young people’s first-ever self-harm, the peak in positive emotions relative to other indicators at the point of self-harm is higher than the peak for positive emotions relative to other indicators for most recent self-harm. This is consistent with previous data from sequential analyses, which reveal an attenuation in feeling positive following self-harm associated with repeat behaviour (30) and which could be associated with habituation (43). Similarly, adult presentations indicated a peak in positive emotions following self-harm for the first-ever episode, which is in marked contrast to the large fall in the SRs for positive emotions following self-harm indicated in the most recent adult self-harm. This pattern is noticeable in an item specifically targeting “feeling better after self-harm,” which clearly shows a stronger endorsement for improvement in feelings post self-harm for the first-ever in comparison to the most recent self-harm in both age groups.

In terms of negative emotion, despite the high frequency with which items were selected by all groups, profiles at individual time points did not reach the notable threshold in the build-up to self-harm, with the exception of the adult’s first ever self-harm which was notable 1 day prior to behaviour. As such, support for negative affect regulation functional accounts of self-harm which hold that individuals are motivated to self-harm to reduce aversive negative emotions receives only partial support in the IWA. We might for example expect to see self-harm preceded by a spike in negative emotions, followed by a reduction in negative mood state, as demonstrated in real-time EMA data (44). There are potential explanations for this. In initial frequency analyses, negative emotion was indeed a high-frequency category and demonstrated a high distribution at all time points prior to and following self-harm, and thus may not have emerged with a greater likelihood of occurrence at any particular time point in IWA. In addition, CaTS permits a highly individualistic representation of self-harm, and consequently, a variety of different forms of negative affect were included within the category.

Different forms of negative affect may have salience across time (45) and it would be helpful to separate out affect presentations in future examinations. In terms of the specific question relating to “feeling worse after self-harm,” response profiles show a clear indication of worsening feelings immediately post-episode for all groups, except again in adult’s first ever self-harm, where feeling worse did not immediately reach the threshold until a later time-point after self-harm. Again, endorsement of feeling worse following self-harm was higher in most recent than the first-ever self-harm for both groups, and by the most recent account of adult self-harm, the absence of feeling worse immediately post self-harm was no longer present. A deeper dive into what “better” and “worse” constitutes could be achieved in card-sort models which zoom into time points, particularly given the complex and multifactorial nature of the emotional response to self-harm. Findings overall underscore a mixed emotional response but suggest a degradation in the function of self-harm as a means of affect regulation, which emerges with repeated self-harm and is relevant for a long time post self-harm.

In terms of developmental differences between young people and adult pathways to self-harm, an interesting distinction is demonstrated in terms of impulsivity, here, specifically the concept of low premeditation or acting on the spur of the moment. This facet of impulsivity is recognised as a risk factor in adolescent and adult self-harm (22, 46) and also plays a prominent role as a key volitional moderator in ideation to enaction models (31), i.e., those who self-harm are more likely to be impulsive than those who have only thought about self-harm (47). The IWA highlights a pattern of an increased likelihood of endorsing impulsivity just before and at the point of self-harm, which is consistent across all groups and which is relevant at the first-ever onset and maintained at the most recent occurrence. These findings extend those from sequence analysis techniques that identify impulsivity as the only immediate precursor to both a first and most recent episode of self-harm to a broader age span (30). Of note, the peak of impulsivity was markedly higher for adolescents at both time points, than for adults, which is in line with previous findings of a developmental difference in levels of impulsivity as associated with self-harm (48) and is consistent with the suggestion that impulsivity is heightened in adolescence (49). Interestingly, premeditation also exceeded the threshold (albeit at a much lower level than impulsivity) for all groups just prior to and at the point of self-harm. Although seemingly at odds, this is consistent with some research that has shown that for some young people a deliberative thought-through plan for self-harm can be delayed if the situation is not suitable, and then subsequently acted on without the need for additional planning (50).

Our data suggested that feelings of isolation, loneliness, and entrapment were marked in pathways for adult self-harm with a notable interdependence 1 h prior to the most recent self-harm. In addition, burdensomeness exceeded the threshold in most recent adult self-harm at 1 week prior to behaviour. This is consistent with recent review evidence suggesting that such factors commonly characterise self-harm in older adults and may be a particular feature of self-harm at an older life stage (17). In fact, loneliness, isolation, and entrapment were similarly interdependent with a time point proximal to self-harm for young people; however, burdensomeness was not and appears to be a particular feature of self-harm at an older age. Interestingly, acquired capability also reached the threshold (at the point of self-harm) for an adult’s most recent self-harm only. Theoretically, it is plausible that an acquired capability to overcome a natural fear of death or pain, would be associated with repeated rather than the first onset of self-harm, and this replicates previous findings (43). Our findings provide temporal evidence consistent with prominent models of suicide and self-harm (31, 33) of the transition through motivational phase moderators (entrapment, burdensomeness, and social isolation) at time points prior to self-harm, which can lead to ideation, to volitional phase moderators (acquired capability) at the point of self-harm, and describing the translation of ideation into action. Our findings indicate that this pathway may be of particular importance in adult behaviour.

Our analyses identified interesting patterns of category selection in relation to accessing support. In terms of support classed as “unhelpful,” both young people and adult pathways demonstrated a sharp increase in accessing unhelpful support immediately after self-harm, peaking later on after their first episode of behaviour. However, accessing unhelpful support did not reach the threshold post self-harm in either case of recent self-harm. Indeed, most recent self-harm behaviours were associated with a sharp rise in accessing support that was classed as “helpful” immediately following self-harm and continued to rise later on after. As such, while there are endorsements for an experience of support that is helpful and unhelpful, there appears to be a different pattern and more positive evaluation of support for more recent presentations. Notably, support access of whatever quality did not feature notably, or reach a nadir, at earlier stages in the timeline. These temporal findings could indicate support for functional motivations of self-harm as a “cry for help” (51), which secures support following self-harm.

It is possible that contemporary help-seeking experiences of young people do qualitatively differ from those of an adult’s first-ever experience of self-harm dating from many years previously. It is also true that there are myriad types of help and support (including informal and formal, online, and face-to-face) and a considerable range in the quality and suitability of this provision, which will differ for individuals and by episode. Recent co-produced avenues of prevention and intervention support, e.g., reaching young audiences by directing support within online social spaces and new media avenues (52, 53) may be contributing to recent improved access and response to support, particularly in young people. Importantly, the findings suggest that groups are help-seeking. There is nonetheless an opportunity to increase targeted support at earlier stages in self-harm pathways, e.g., through universal prevention programmes. Importantly, the broad categorisation of helpful or unhelpful support also masks the salience of individual support-related items. Inspection of frequencies of card selection (Supplementary material 1) reveals that items relating to formal support seeking through dedicated services such as GPs or teachers were endorsed less frequently than informal sources of support such as friends and partners. Increased participatory approaches would support an improved understanding of barriers and facilitators to support seeking and engagement and improve the design and implementation of service provision. For example, recent work focused on improving the experience of visiting a GP and preparing young people for their GP consultation (54) models how youth-oriented involvement and co-production can facilitate improved formal service use.

## Limitations

There are limitations associated with the nature of the CaTS-task itself which relies on retrospective recall. It is recognised that for adult participants, in particular, this may have required recalling an episode of self-harm occurring many years previously, and as such the accuracy of recall, in particular, in terms of the placing of items against a pinpointed timeframe, may be open to question. Having said this, the first-ever experience of self-harm is a focal event that is likely to be subject to easier recall than other experiences. While caution is advocated in the interpretation of findings as discussed, responses provided show consistent parallels with key theoretical frameworks. There were also commonalities occurring across groups indicate a shared pattern of behaviour. It is noteworthy nonetheless that overall adults selected half the number of cards that younger participants selected, which could indicate difficulties with recall. Lower card usage by adults could also relate to reluctance or difficulty engaging with the online tool. In addition, the online and anonymous nature of the CaTS-online tool limited the ability to understand reasons for failure to complete the task (e.g., lack of interest, not being able to remember details, lack of understanding, and wanting to exit the task) and precluded the ability to sense check with participants. This limitation could be addressed through a more collaborative digital implementation, e.g., within a therapeutic session.

While our aim was to identify similarities and differences between self-harm in young people and adults and hence we distinguished groups at a cut-off of 25 years in accordance with established categorisations of adolescence (2), this may nonetheless represent fairly arbitrary segregation at a developmental level and as such our groups may not be sufficiently distinct. In the vast majority of cases, adult participants also indicated an adolescent first incidence of self-harm, and as such, adult first ever and young person first ever were both representations of self-harm onset pre-adulthood. However, it is also important to recognise that contemporary risk factors, attitudes towards, experiences of support for self-harm, etc., are likely to differ considerably between these groups at the time of the first incidence. For example, young people today are living in extraordinarily stressful times, with unprecedented social and online pressure, increased psychological distress, and high levels of self-dissatisfaction (10), particularly among girls. A larger or more targeted sampling frame could allow for the comparison of adolescent/young person incident vs. adult incident self-harm.

There are also limitations to discuss in relation to the IWA approach as employed to analyse CaTS data. Grouping cards into categories in the first stage of IWA facilitates relatively simple data outputs for the ease of analysis and presentation but necessarily removes some of the complexity and nuance associated with the multi-card task. In some cases, related concepts were included in a shared category (e.g., loneliness, isolation, and entrapment) which reduces their specificity. Arguably, the IWA can be used to pinpoint areas that warrant examination at greater granulation. It would be informative to explore in more detail the importance of individual items that show variable prominence at time points or age groups within a multi-item category. A strength of the original CaTS development was the involvement of young people with lived experience in the identification of card items. Continued development of the CaTS would benefit from further co-production and expert involvement work to refine and update item selection, potential category membership, and analysis approach. An advantage of IWA is that participants were able to use cards multiple times. In some cases, this resulted in multiple uses of the same card (e.g., “I felt depressed and sad”) at every time point preceding self-harm, and thus IWA only showed limited notable interdependence for the category of negative emotion at time points. It is also possible that low mood is an ever-present indicator leading to self-harm, which is not notable at any one particular time point. An additional limitation of the approach as applied to self-harm items in this study was the inclusion of two cards that included a time reference (feeling positive after self-harm or feeling negative after self-harm) that were not, therefore, independent of timing. It is, however, possible to draw comparisons on the selection of either card across groups. Finally, it should be noted that young participants were offered participation in exchange for course credit which may have introduced incentivised bias.

Creating CaTS-online brought benefits in terms of increasing reach and access to a large community-based sample and increased functionality (i.e., being able to select a card multiple times), but there are ethical limitations with a task that explores complex and sensitive topics in a detailed approach, remotely, and outside of support. Factors were included to mitigate risk, including an 18+ age range, and signposting. Nonetheless, we report a decline in VAS scores at the completion of the task though this was significant for young people only. In fact, this finding is consistent with other studies that have reported a reduction in mood following participation in self-harm research, but which have also indicated that changes are often short-lived and while the mood is impacted, this is not necessarily judged as distressing (55, 56).

## Implications and next steps

### Research implications

• Our findings offer support for prominent theories and models, including the Interpersonal Theory of Suicidal Behaviour e.g., (33) and the IMV (31), and contribute to an understanding of the relative interplay between multiple factors associated with risk for self-harm over a period of time. By also comparing pathways between the first-ever and most recent self-harm, we identify the patterns that emerge over repeated behaviour, including notably changed experiences of support. Further work delineating factors associated with “helpful” or “unhelpful” support at stages of the self-harm pathway are particular points of note resulting from this work.

• We address calls for greater representation of community-based adult samples (17) and point to commonalities and differences in the characteristic pattern of indicators noted for adults and young people, suggesting that some indicators, e.g., the nature of belonging for young people or the nature of burdensomeness for adults are of heightened relevance prior to self-harm (relative to other multiple and shared risk factors). The characteristic profiling approach adopted here speaks to wider approaches that seek to delineate distinctive psychological risk profiles [e.g., (57)] in order to provide more targeted treatment options throughout the self-harm cycle.
• The adoption of IWA supports calls for innovative approaches and techniques to account for the complexity and dynamic nature of self-harm (29). Using IWA with a card-sort task is novel, and we look now to refine and improve this process. The IWA does not provide a prediction of behaviour, but rather a “characteristic signature” visible at time points in relation to self-harm, for example, a week before. Such an approach may support the early identification of “warnings signs” to be picked up and monitored. However, turning the patterns of peaks and troughs into a statistically valid prediction (given the measure is derived from standard residuals rather than raw frequencies) is something still being explored.

### Clinical implications

• In line with previous CaTS examinations, we identify what factors might be indicators of risk (such as increased impulsivity) and an understanding of when in relation to other complex factors these factors are likely to become most salient. As such, the findings offer targeted and time-specific points for intervention. Many of the factors identified are modifiable and exist as treatment targets within therapeutic approaches (e.g., DBT-A). The use of a tool such as CaTS within a clinical setting could support clinicians with a needs-based assessment in line with recently published NICE guidelines (58) and supports decision-making on the timing and pertinence of therapeutic support. In addition, there may be value in the mapping of individual risk profiles *via* visual diagrams which are simple to generate, readable, and can support collaborative discussion and shared understanding between patients and practitioners within and across care settings.

### Next steps

• This study used a prototype version of CaTS-online and provides a pilot test of this initial digital iteration of CaTS. Developing the task into a digital innovation was anecdotally discussed within our PPI group. As a first test, the study shows at a practical level that participants were able to engage with and complete the task in this format, and the extracted data was sufficient for analyses. As such, the further development of CaTS as a digital tool (e.g., an online version or a mobile app) merits exploration. Work is now needed to explore in greater depth how that tool might look, function, or be implemented, and build an understanding of acceptability, usability, and feasibility. As part of this, we might revisit the cards and card categories in the original CaTS to consider new markers of risk and how to group the cards. Such work should be driven by a strong co-production focus (59).

## Conclusion

The CaTS offers a systematic approach to understanding the complex interplay between thoughts, feelings, events, and behaviours in the build-up to self-harm. A pilot test of CaTS-online using a novel analytical approach (IWA) successfully captured and visually presented the multidimensional and dynamic pattern of risk across young people and adults’ first-ever and most recent pathways. Key indicators of risk and intervention points are identified and indicate the changing profile of risk with repeat self-harm. Work to extend these pilot findings should further develop the potential for digital application, particularly, in clinical settings.

## Data availability statement

Due to the sensitive nature of this topic, supporting data is available with conditions. Further information about the data and conditions for access are available at the University of Nottingham data repository (doi: 10.17639/nott.72).

## Ethics statement

The studies involving human participants were reviewed and approved by University of Nottingham, School of Psychology Research Ethics Committee. The patients/participants provided their written informed consent to participate in this study.

## Author contributions

IT and ET conceptualised and designed the study. AB and IT developed the CaTS-online with an undergraduate research team. IT recruited the participants and performed the data collection alongside an undergraduate research team. CB and KB performed the analysis. JL wrote the first draft of the manuscript. JL, CB, IT, KB, and ET were responsible for interpreting results. DC devised the original CaTS and Indicator Waves techniques as part of an earlier project on self-harm led by ET. All authors contributed to preparing and approving the final manuscript.
