# Supplementary material for: A comparison of temporal pathways to self-harm in young people compared to adults: A pilot test of the Card Sort Task for Self-harm online using Indicator Wave Analysis
Source: Front Psychiatry. 2023 Jan 12;13:938003. doi: 10.3389/fpsyt.2022.938003 (PMC9878399; doi:10.3389/fpsyt.2022.938003)
Supplement: Supplementary file 2 [file Table_2.DOCX]

S2

Cards grouped based on the literature to form categories for Indicator Wave Analysis

| **Decision Making and Judgement** |
| --- |
| A05 |
| A06 |
| A08 |
| B24 |
| **Premeditation** |
| D11 |
| **Acquired capability** |
| A02 |
| D08 |
| D18 |
| **Impulsivity** |
| D15 |
| **Negative Life events or social problems** |
| C01 |
| C02 |
| C03 |
| C04 |
| C05 |
| C06 |
| C07 |
| C09 |
| C15 |
| C16 |
| C18 |
| C19 |
| C20 |
| C21 |
| C22 |
| C23 |
| C24 |
| C25 |
| C26 |
| C28 |
| D06 |
| D07 |
| D16 |
| D17 |
| **Loneliness, entrapment and isolation** |
| A04 |
| B14 |
| B16 |
| B20 |
| B21 |
| D12 |
| **Exposure** |
| C08 |
| C10 |
| C11 |
| C12 |
| C13 |
| C27 |
| **Negative Emotions** |
| B01 |
| B04 |
| B05 |
| B06 |
| B07 |
| B10 |
| B26 |
| **Humiliation and Defeat** |
| B19 |
| B15 |
| **Positive Emotions** |
| B09 |
| **Feeling negative after self-harm** |
| F02 |
| **Feeling Positive after self harm** |
| F06 |
| **Accessed Support that didn't help** |
| E03 |
| E05 |
| E07 |
| E09 |
| E11 |
| E17 |
| E19 |
| E23 |
| E25 |
| F05 |
| **Accessed support that helped** |
| A12 |
| E01 |
| E02 |
| E04 |
| E06 |
| E08 |
| E10 |
| E16 |
| E18 |
| E20 |
| E21 |
| E22 |
| E24 |
| F04 |
| **Lack of support** |
| A03 |
| A09 |
| A10 |
| C17 |
| **Burdensomeness** |
| B17 |
| **Lack of belonging** |
| B22 |
| B23 |
| C14 |
