# Supplementary material for: A comparison of temporal pathways to self-harm in young people compared to adults: A pilot test of the Card Sort Task for Self-harm online using Indicator Wave Analysis
Source: Front Psychiatry. 2023 Jan 12;13:938003. doi: 10.3389/fpsyt.2022.938003 (PMC9878399; doi:10.3389/fpsyt.2022.938003)
Supplement: Supplementary file 3 [file Table_3.DOCX]

S3

Other self-reported methods of self-harm in addition to self-injury and/or self-poisoning.

| Additional reported methods |
| --- |
| Crashing car with intention to harm oneself (1)  Bloodletting (2)  Unsafe sex (1)  Alcohol/drugs (1)  Purging (3)  Eating disorder (4)  Self-induced vomiting (1)  Scratching (1)  Hair pulling (2)  Skin picking (1)  Interfering with wounds (1)  Beating body with fists (1) |

N.B. Some participants contributed more than one additional method of self-harm to this list.
