# Supplementary material for: A comparison of temporal pathways to self-harm in young people compared to adults: A pilot test of the Card Sort Task for Self-harm online using Indicator Wave Analysis
Source: Front Psychiatry. 2023 Jan 12;13:938003. doi: 10.3389/fpsyt.2022.938003 (PMC9878399; doi:10.3389/fpsyt.2022.938003)
Supplement: Supplementary file 4 [file Table_4.DOCX]

S4

The total frequency of individual cards occurring across the timeline for each group and the description of each card within the card set.

|  |  |  | Total Frequency | | | |
| --- | --- | --- | --- | --- | --- | --- |
| Card sets | Card | Card Description | Adult | | Young People | |
|  |  |  | First Ever | Most Recent | First Ever | Most Recent |
| Thoughts | A01 | I wanted to die | 32 | 28 | 77 | 67 |
|  | A02 | I was not afraid of death | 17 | 10 | 50 | 33 |
|  | A03 | There was no one to turn to for help | 32 | 30 | 89 | 35 |
|  | A04 | I could not trust anyone | 29 | 11 | 44 | 20 |
|  | A05 | I struggled to make decisions | 12 | 16 | 26 | 29 |
|  | A06 | I could not think of anything else to do | 12 | 18 | 52 | 32 |
|  | A07 | I wanted to kill myself | 15 | 18 | 37 | 33 |
|  | A08 | I could not solve a problem I faced | 8 | 24 | 28 | 19 |
|  | A09 | I could not tell anyone how I was feeling | 48 | 38 | 84 | 50 |
|  | A10 | No one listened to me or took me seriously | 12 | 7 | 37 | 13 |
|  | A11 | I thought about being very badly treated as a young child | 3 | 10 | 35 | 20 |
|  | A12 | I trusted a caregiver | 1 | 0 | 0 | 0 |
|  | A13 | I had flashbacks about something bad that happened | 5 | 24 | 28 | 26 |
| Feelings | B01 | I was angry | 22 | 34 | 56 | 45 |
|  | B02 | I felt I would not be able to change myself in the future | 16 | 20 | 27 | 31 |
|  | B03 | The mental pain was unbearable | 26 | 39 | 71 | 42 |
|  | B04 | I felt depressed and sad | 53 | 42 | 132 | 99 |
|  | B05 | I felt very anxious | 24 | 42 | 81 | 61 |
|  | B06 | I felt worthless | 29 | 32 | 96 | 59 |
|  | B07 | I felt disgusting | 16 | 16 | 57 | 29 |
|  | B08 | I felt exhausted | 11 | 36 | 56 | 47 |
|  | B09 | I felt energized | 5 | 8 | 10 | 14 |
|  | B10 | I hated myself | 47 | 54 | 104 | 73 |
|  | B11 | I felt hopeful about the future | 3 | 3 | 1 | 3 |
|  | B12 | I felt I could change for the better in the future | 0 | 4 | 3 | 4 |
|  | B13 | I felt I could not change for the better in the future | 3 | 13 | 18 | 14 |
|  | B14 | I felt trapped | 35 | 29 | 56 | 29 |
|  | B15 | I felt defeated | 9 | 19 | 43 | 40 |
|  | B16 | I felt I could not escape from feelings or situations | 22 | 25 | 46 | 49 |
|  | B17 | I felt like a burden on people | 6 | 9 | 48 | 31 |
|  | B18 | I felt very hopeless about the future | 24 | 19 | 33 | 44 |
|  | B19 | I felt humiliated | 6 | 3 | 23 | 11 |
|  | B20 | I felt like I did not belong | 36 | 7 | 39 | 25 |
|  | B21 | I felt ignored | 9 | 3 | 23 | 17 |
|  | B22 | I felt rejected | 8 | 9 | 27 | 12 |
|  | B23 | I felt abandoned | 5 | 6 | 19 | 14 |
|  | B24 | I did not know what I was feeling | 23 | 12 | 15 | 3 |
|  | B25 | I felt numb | 9 | 18 | 60 | 32 |
|  | B26 | I felt ashamed | 18 | 25 | 39 | 28 |
| Events | C01 | I was being abused physically | 5 | 3 | 18 | 0 |
|  | C02 | I was being abused mentally | 10 | 7 | 43 | 2 |
|  | C03 | I had an argument with my friend | 2 | 2 | 8 | 4 |
|  | C04 | I had an argument with my boyfriend/girlfriend | 1 | 5 | 10 | 15 |
|  | C05 | I had an argument with my parent/caregiver | 5 | 0 | 13 | 5 |
|  | C06 | I got into trouble with the police | 0 | 0 | 4 | 2 |
|  | C07 | I was raped | 5 | 5 | 6 | 3 |
|  | C08 | I knew someone who was self-harming | 7 | 4 | 44 | 2 |
|  | C09 | I was a victim of a crime | 4 | 2 | 3 | 2 |
|  | C10 | Someone I knew killed themselves | 1 | 2 | 3 | 1 |
|  | C11 | Lots of people I knew were doing it | 2 | 1 | 11 | 2 |
|  | C12 | I read about self-harm on the internet | 4 | 1 | 23 | 7 |
|  | C13 | I discussed self-harm in a forum on the internet | 1 | 6 | 4 | 1 |
|  | C14 | I was rejected by my parents | 2 | 0 | 7 | 2 |
|  | C15 | I was taken into foster care | 0 | 0 | 0 | 0 |
|  | C16 | I was taken into a residential care home | 0 | 0 | 0 | 0 |
|  | C17 | I received no support from caregivers | 5 | 1 | 9 | 0 |
|  | C18 | I was bullied | 16 | 4 | 38 | 2 |
|  | C19 | I had a problem at school | 12 | 5 | 24 | 4 |
|  | C20 | I had a problem at work | 3 | 12 | 0 | 7 |
|  | C21 | Someone close to me died | 1 | 5 | 8 | 3 |
|  | C22 | I moved to a different home | 2 | 5 | 2 | 7 |
|  | C23 | Someone close to me left | 0 | 1 | 4 | 8 |
|  | C24 | I moved to a different school | 3 | 0 | 3 | 2 |
|  | C25 | I was having problems in a close relationship | 4 | 11 | 7 | 9 |
|  | C26 | I had a problem at university/college | 1 | 11 | 4 | 12 |
|  | C27 | Someone I knew tried to kill themselves | 0 | 0 | 6 | 2 |
|  | C28 | My home life was not very good | 16 | 2 | 31 | 19 |
| Behaviours | D01 | I was very agitated and restless | 16 | 23 | 35 | 40 |
|  | D02 | I was drunk | 5 | 3 | 7 | 12 |
|  | D03 | I was high on drugs | 1 | 0 | 3 | 9 |
|  | D04 | I had unprotected sex | 1 | 0 | 2 | 5 |
|  | D05 | I was not able to sleep | 18 | 22 | 42 | 32 |
|  | D06 | I was having nightmares | 1 | 9 | 29 | 25 |
|  | D07 | I got involved with a gang | 0 | 0 | 0 | 0 |
|  | D08 | I am insensitive to pain | 7 | 5 | 10 | 2 |
|  | D09 | I was drinking alcohol | 5 | 15 | 19 | 12 |
|  | D10 | I was taking illegal drugs | 4 | 0 | 14 | 5 |
|  | D11 | I planned it carefully | 5 | 19 | 14 | 5 |
|  | D12 | I isolated myself from others | 34 | 37 | 53 | 32 |
|  | D13 | I did other things to hurt myself (starved myself, drank too much) | 17 | 27 | 42 | 23 |
|  | D14 | I had access to the means to hurt myself | 19 | 27 | 40 | 35 |
|  | D15 | I did it on impulse without planning | 23 | 8 | 32 | 25 |
|  | D16 | I got into trouble at school/work | 2 | 3 | 9 | 6 |
|  | D17 | I got into trouble at home | 7 | 1 | 6 | 1 |
|  | D18 | I am used to pain | 5 | 11 | 10 | 14 |
| Services and support | E01 | Someone listened to me and took me seriously | 1 | 10 | 6 | 9 |
|  | E02 | I received therapy which helped | 2 | 9 | 5 | 10 |
|  | E03 | I received therapy which did not help | 2 | 5 | 12 | 5 |
|  | E04 | I talked to a friend which helped | 3 | 7 | 11 | 17 |
|  | E05 | I talked to a friend which did not help | 9 | 4 | 11 | 7 |
|  | E06 | I talked to my caregivers which helped | 1 | 0 | 1 | 1 |
|  | E07 | I talked to my caregivers which did not help | 1 | 2 | 6 | 1 |
|  | E08 | I saw my GP which helped | 2 | 5 | 2 | 4 |
|  | E09 | I saw my GP which did not help | 3 | 5 | 4 | 6 |
|  | E10 | I phoned a helpline which helped | 0 | 0 | 1 | 0 |
|  | E11 | I phoned a helpline which did not help | 0 | 3 | 5 | 8 |
|  | E12 | I went to counselling which helped | 0 | 2 | 6 | 4 |
|  | E13 | I went to counselling which did not help | 2 | 2 | 6 | 2 |
|  | E14 | I read a self-help book – it helped | 0 | 1 | 0 | 1 |
|  | E15 | I read a self-help book – it did not help | 0 | 2 | 1 | 1 |
|  | E16 | I talked to a teacher which helped | 3 | 0 | 3 | 2 |
|  | E17 | I talked to a teacher which did not help | 3 | 0 | 4 | 0 |
|  | E18 | I talked to a social worker which helped | 0 | 0 | 0 | 0 |
|  | E19 | I talked to a social worker which did not help | 0 | 0 | 1 | 0 |
|  | E20 | I received help and support from a user-led service (e.g. Harmless) | 0 | 2 | 1 | 0 |
|  | E21 | I depended on a caregiver for help and support | 0 | 0 | 0 | 1 |
|  | E22 | I talked to a boyfriend/girlfriend which helped | 1 | 5 | 10 | 15 |
|  | E23 | I talked to a boyfriend/girlfriend which did not help | 0 | 1 | 7 | 3 |
|  | E24 | I talked to a mental health support worker which helped | 0 | 2 | 1 | 4 |
|  | E25 | I talked to a mental health support worker which did not help | 2 | 6 | 2 | 4 |
| Afterwards | F01 | I went to hospital for overdose or self-injury | 3 | 5 | 9 | 10 |
|  | F02 | I felt worse after self-harm | 5 | 9 | 13 | 20 |
|  | F03 | Self-harm stopped me from killing myself | 7 | 8 | 7 | 5 |
|  | F04 | A & E staff were friendly and understanding | 1 | 4 | 1 | 3 |
|  | F05 | A & E staff were not friendly and understanding | 1 | 2 | 2 | 1 |
|  | F06 | I felt better after self-harm | 35 | 16 | 43 | 23 |
|  | F07 | I felt no different after self-harm | 7 | 12 | 17 | 12 |
