# Supplementary material for: A comparison of temporal pathways to self-harm in young people compared to adults: A pilot test of the Card Sort Task for Self-harm online using Indicator Wave Analysis
Source: Front Psychiatry. 2023 Jan 12;13:938003. doi: 10.3389/fpsyt.2022.938003 (PMC9878399; doi:10.3389/fpsyt.2022.938003)
Supplement: Supplementary file 5 [file Table_5.DOCX]

S5 Indicator Waves Frequency Matrix

SRs are highlighted in green or red if they meet a threshold value of ≥ 2.0, indicating higher or lower frequency than would be expected by chance, respectively.

## Adult (First Ever)

|  | **Adult (First Ever)** | | | | | | | | | |
| --- | --- | --- | --- | --- | --- | --- | --- | --- | --- | --- |
|  | **[+6Months]** | **[6Months]** | **[1Month]** | **[1Week]** | **[1Day]** | **[1Hour]** | **[JustBefore]** | **[SelfHarm]** | **[Immediately After]** | **[LongerAfter]** |
| Decision Making and Judgment | -1 | -1 | 1.1 | -0.6 | -1.6 | 0.6 | 2.2 | 0.2 | 0.6 | -0.8 |
| Premeditation | -0.8 | -0.7 | -0.7 | 0.8 | 1 | 2 | 0.4 | -0.7 | -0.6 | -0.7 |
| Acquired capability | -1.5 | -0.4 | -0.4 | 0.1 | -0.1 | 1 | 0 | 1.4 | 0.1 | 0 |
| Impulsivity | -1.7 | -1.5 | -1.5 | -1.5 | -1.3 | -0.3 | 3.9 | 5.3 | -0.4 | -1.5 |
| Negative Life events or social problems | 5.7 | 1.6 | 1.5 | 0.2 | 0.5 | 0 | -1.8 | -2.7 | -2.6 | -3.2 |
| Loneliness, entrapment and isolation | 0.9 | 1.3 | 1.4 | 0.8 | -0.1 | 0 | 0.3 | -0.9 | -1.8 | -2.2 |
| Exposure | -1.4 | 3.1 | 3 | 0.5 | -0.1 | 0.3 | -1.4 | -1.2 | -1 | -1.2 |
| Humiliation and Defeat | -0.7 | -0.3 | -1.2 | 0.5 | -1.1 | 0.3 | 0 | 2.2 | 0 | 0.4 |
| Positive Emotions | -0.8 | -0.7 | -0.7 | -0.7 | -0.6 | -0.7 | 0.4 | 2.2 | 2.9 | -0.7 |
| Feeling Positive after self harm | -1.7 | -1.8 | -1.8 | -1.8 | -1.6 | -2 | -2.2 | -1.3 | 14.3 | 2.8 |
| Feeling negative after self-harm | -0.8 | -0.7 | -0.7 | -0.7 | -0.6 | -0.7 | -0.8 | -0.7 | -0.6 | 6.2 |
| Negative Emotions | -2.1 | -0.9 | -0.4 | 0.7 | 2.6 | 0.8 | 1.2 | 0.5 | -2.1 | -0.4 |
| Accessed Support that didn't help | -0.5 | 0.8 | -1.4 | -1.4 | -1.3 | -1.5 | -1.7 | -1.4 | -0.3 | 8.7 |
| Accessed support that helped | -1.4 | -1.2 | -1.2 | -1.2 | -1.1 | -0.5 | -1.4 | -1.2 | -1 | 10 |
| Lack of support | 1.1 | 0.4 | -0.4 | 0.9 | 0.6 | -0.2 | -1.2 | 1 | -0.2 | -1.9 |
| Burdensomeness | 1.3 | 0.6 | -0.8 | -0.8 | 0.8 | -0.8 | -0.9 | 0.6 | -0.6 | 0.5 |
| Lack of belonging | 0.6 | -0.4 | -0.4 | 0.4 | -1.1 | 0.2 | 1.9 | -0.4 | -0.1 | -1.3 |

## Adult (Most Recent)

|  | **Adult (Most Recent)** | | | | | | | | | |
| --- | --- | --- | --- | --- | --- | --- | --- | --- | --- | --- |
|  | **[+6Months]** | **[6Months]** | **[1Month]** | **[1Week]** | **[1Day]** | **[1Hour]** | **[JustBefore]** | **[SelfHarm]** | **[Immediately After]** | **[LongerAfter]** |
| Decision Making and Judgment | -0.5 | -0.1 | -0.8 | -1.1 | 1.2 | 1 | 1.2 | 1.4 | -1 | -1.8 |
| Premeditation | -1 | -1.3 | -0.7 | 1.6 | 2.3 | 0.7 | 0.9 | -0.6 | -1.2 | -1.2 |
| Acquired capability | -0.8 | -0.9 | -0.4 | -1 | -0.3 | 0.2 | 0.8 | 2.9 | 0 | -0.6 |
| Impulsivity | -1 | -0.8 | -0.9 | -0.9 | -0.9 | -0.9 | 1.9 | 4.9 | -0.8 | -0.7 |
| Negative Life events or social problems | 6 | 2.4 | 2.1 | 0.7 | 0.3 | -2.5 | -2.1 | -2.9 | -2.6 | -2.6 |
| Loneliness, entrapment and isolation | -1.6 | -0.3 | 0.2 | 1.6 | 0.6 | 2.4 | 0.3 | -1.4 | -0.1 | -1.7 |
| Exposure | 2.2 | 0.7 | 0.5 | -0.3 | 0.5 | -0.4 | -0.6 | -1.1 | -1 | -1 |
| Humiliation and Defeat | -1.2 | -0.7 | 1.8 | -0.8 | -0.8 | -0.9 | 0.6 | 1.4 | -0.5 | 1.2 |
| Positive Emotions | -1 | -0.8 | -0.9 | -0.9 | -0.9 | -0.9 | -0.1 | 7.2 | -0.8 | -0.7 |
| Feeling Positive after self harm | -1.5 | -1.2 | -1.3 | -1.2 | -1.3 | -1.3 | -1.5 | -1.2 | 9.9 | 2.7 |
| Feeling negative after self-harm | -1.1 | -0.9 | -1 | -0.9 | -0.9 | -1 | -1.1 | -0.9 | 4 | 5.5 |
| Negative Emotions | -0.9 | 0.7 | -0.2 | 0.5 | -0.2 | 1.2 | 1.1 | 0 | -1 | -1.5 |
| Accessed Support that didn't help | 0.6 | 1.6 | 1.3 | 0.8 | 0.1 | -1.1 | -1.9 | -0.4 | -0.1 | -0.7 |
| Accessed support that helped | 0.4 | -1 | -1.2 | -1.1 | -2.1 | -2.2 | -2.4 | -2 | 1.5 | 12.5 |
| Lack of support | -0.4 | -1 | 0.1 | -2 | 0.6 | 1.4 | 0.6 | 1.1 | 0.5 | -1 |
| Burdensomeness | -1.1 | 0.2 | -1 | 3.3 | 0.1 | -1 | 0.7 | -0.9 | 0.4 | -0.8 |
| Lack of belonging | 0 | -0.3 | -0.4 | 1.3 | -0.4 | -0.5 | 1.4 | -0.3 | -0.1 | -1 |

## Young People (First Ever)

|  | **Young People (First Ever)** | | | | | | | | | |
| --- | --- | --- | --- | --- | --- | --- | --- | --- | --- | --- |
|  | **[+6Months]** | **[6Months]** | **[1Month]** | **[1Week]** | **[1Day]** | **[1Hour]** | **[JustBefore]** | **[SelfHarm]** | **[Immediately After]** | **[LongerAfter]** |
| Decision Making and Judgment | -0.7 | -1.2 | 0.1 | -1.3 | 1.3 | 1.3 | 1.7 | 2 | -1.3 | -1.6 |
| Premeditation | -1.5 | -1.2 | -0.6 | 0.2 | 1.5 | 1.7 | 2.5 | -1.1 | 0.3 | -1.1 |
| Acquired capability | -0.5 | -0.8 | -0.3 | -0.2 | 1 | 0.9 | 0.8 | 0.6 | 0.1 | -1.2 |
| Impulsivity | -1.4 | -1.8 | -1.5 | -2 | 0 | -1 | 3.1 | 9 | -1.3 | -1.6 |
| Negative Life events or social problems | 6.4 | 2.2 | 0.3 | 1.6 | 0.4 | -2.2 | -3 | -3.1 | -3 | -3.5 |
| Loneliness, entrapment and isolation | -0.8 | 0.3 | 0.8 | 1.5 | -0.1 | 0.3 | 2 | 0.3 | -2.4 | -2.5 |
| Exposure | 0.5 | 1.9 | 1.3 | 1.1 | 1.2 | -1.3 | -1.4 | -2 | -2.2 | -0.6 |
| Humiliation and Defeat | -1.2 | -0.7 | 0.5 | -0.4 | -0.5 | -1 | 0.5 | 1.2 | 1.8 | 0.6 |
| Positive Emotions | -1.3 | -1 | -1.1 | -1.1 | -1 | 1.3 | 1.2 | 4.7 | 0.6 | -0.9 |
| Feeling Positive after self harm | -2.3 | -2.1 | -2.3 | -2.3 | -2 | -1.9 | -1.9 | 1.4 | 18.2 | 1.3 |
| Feeling negative after self-harm | -1.5 | -1.2 | -1.3 | -1.3 | -1.1 | -1 | -1.1 | -1 | 5.1 | 6.6 |
| Negative Emotions | -1.6 | 0.2 | -0.2 | -0.6 | 0.9 | 1.8 | 1.4 | 1.2 | -1 | -1.4 |
| Accessed Support that didn't help | 0.3 | -0.6 | -1.5 | 0.1 | -1.4 | -2.1 | -2.2 | -2.1 | -0.5 | 10.1 |
| Accessed support that helped | 0.8 | 1.2 | 1.7 | 1.7 | -0.5 | 1.9 | -0.9 | -2.7 | -2 | -2.6 |
| Lack of support | -1.1 | -0.9 | 1.2 | -1.2 | 0.2 | 0 | -0.6 | 0.6 | 0.9 | 1.5 |
| Burdensomeness | -1.1 | -0.9 | 1.2 | -1.2 | 0.2 | 0 | -0.6 | 0.6 | 0.9 | 1.5 |
| Lack of belonging | 1 | -0.2 | 1.2 | -0.7 | -0.9 | 0.3 | 0.6 | -0.6 | -0.5 | -0.7 |

## Young People (Most Recent)

|  | **Young People (Most Recent)** | | | | | | | | | |
| --- | --- | --- | --- | --- | --- | --- | --- | --- | --- | --- |
|  | **[+6Months]** | **[6Months]** | **[1Month]** | **[1Week]** | **[1Day]** | **[1Hour]** | **[JustBefore]** | **[SelfHarm]** | **[Immediately After]** | **[LongerAfter]** |
| Decision Making and Judgment | 1.5 | 0.5 | -0.6 | 0 | 0.1 | 1 | 0.9 | -0.4 | -2.1 | -1.9 |
| Premeditation | -0.8 | -0.7 | -0.8 | -0.7 | 0.7 | 1.6 | -0.7 | 2.4 | -0.6 | -0.6 |
| Acquired capability | 1 | 0.3 | 0.4 | -1.3 | -0.5 | -0.3 | 1.1 | 0.9 | -0.9 | -1.2 |
| Impulsivity | -1.7 | -1.5 | -1.7 | -0.9 | -1 | -1.3 | 0.7 | 10.9 | -1.4 | -1.3 |
| Negative Life events or social problems | 3.6 | 0.7 | 2 | 2.1 | 0.9 | -0.3 | -2.1 | -2.8 | -2.6 | -3 |
| Loneliness, entrapment and isolation | -0.6 | 0.4 | 0.9 | 0.8 | 0.2 | 0.6 | 1.6 | -0.9 | -1.6 | -2.4 |
| Exposure | 1 | 0.5 | -1.3 | 1.3 | 0.3 | 0.6 | -1.3 | 0.7 | -1.1 | -1 |
| Humiliation and Defeat | -0.3 | -0.8 | 0.3 | -0.4 | -0.6 | -0.1 | 0.6 | 0.3 | 0.6 | 0.4 |
| Positive Emotions | -0.5 | -1.1 | -1.3 | -1.2 | 0.4 | -0.7 | 0.4 | 2.6 | 1.9 | 0.1 |
| Feeling Positive after self harm | -1.6 | -1.4 | -1.7 | -1.5 | -1.6 | -1.8 | -1.6 | 0.8 | 10.1 | 2.9 |
| Feeling negative after self-harm | -1.5 | -1.3 | -1.6 | -1.4 | -1.4 | -1.1 | -1.5 | -0.5 | 8.6 | 4.1 |
| Negative Emotions | -2.2 | -0.8 | 0.5 | 0.8 | 0.4 | 0.6 | 0.8 | 0.5 | 0.1 | -1.1 |
| Accessed Support that didn't help | 2 | 2.7 | -0.6 | -0.7 | 0.2 | -1.3 | -0.4 | -1.1 | -1 | 0.5 |
| Accessed support that helped | 0 | -1.3 | -2.1 | -1.8 | -2.3 | -2 | -2.4 | -2.1 | 2.6 | 15 |
| Lack of support | 0.3 | 1.7 | 0.9 | -0.1 | 1.2 | 0.3 | -0.9 | -1.5 | -1.2 | -1.3 |
| Burdensomeness | -0.3 | 1.3 | 0.6 | 0.6 | -0.1 | -0.2 | 0.3 | -1 | -1.5 | 0 |
| Lack of belonging | -0.2 | -1 | -0.8 | -1.1 | 0.6 | 2.4 | 1.6 | -0.3 | -0.8 | -1.4 |
