# Supplementary material for: A comparison of temporal pathways to self-harm in young people compared to adults: A pilot test of the Card Sort Task for Self-harm online using Indicator Wave Analysis
Source: Front Psychiatry. 2023 Jan 12;13:938003. doi: 10.3389/fpsyt.2022.938003 (PMC9878399; doi:10.3389/fpsyt.2022.938003)

S6 Indicator Wave graph showing the temporal organisation of categories across time points for Adult's and Young People’s first ever and most recent episode of self-harm. Standardised residuals +/- 2 indicating higher or lower frequency than would be expected by chance.
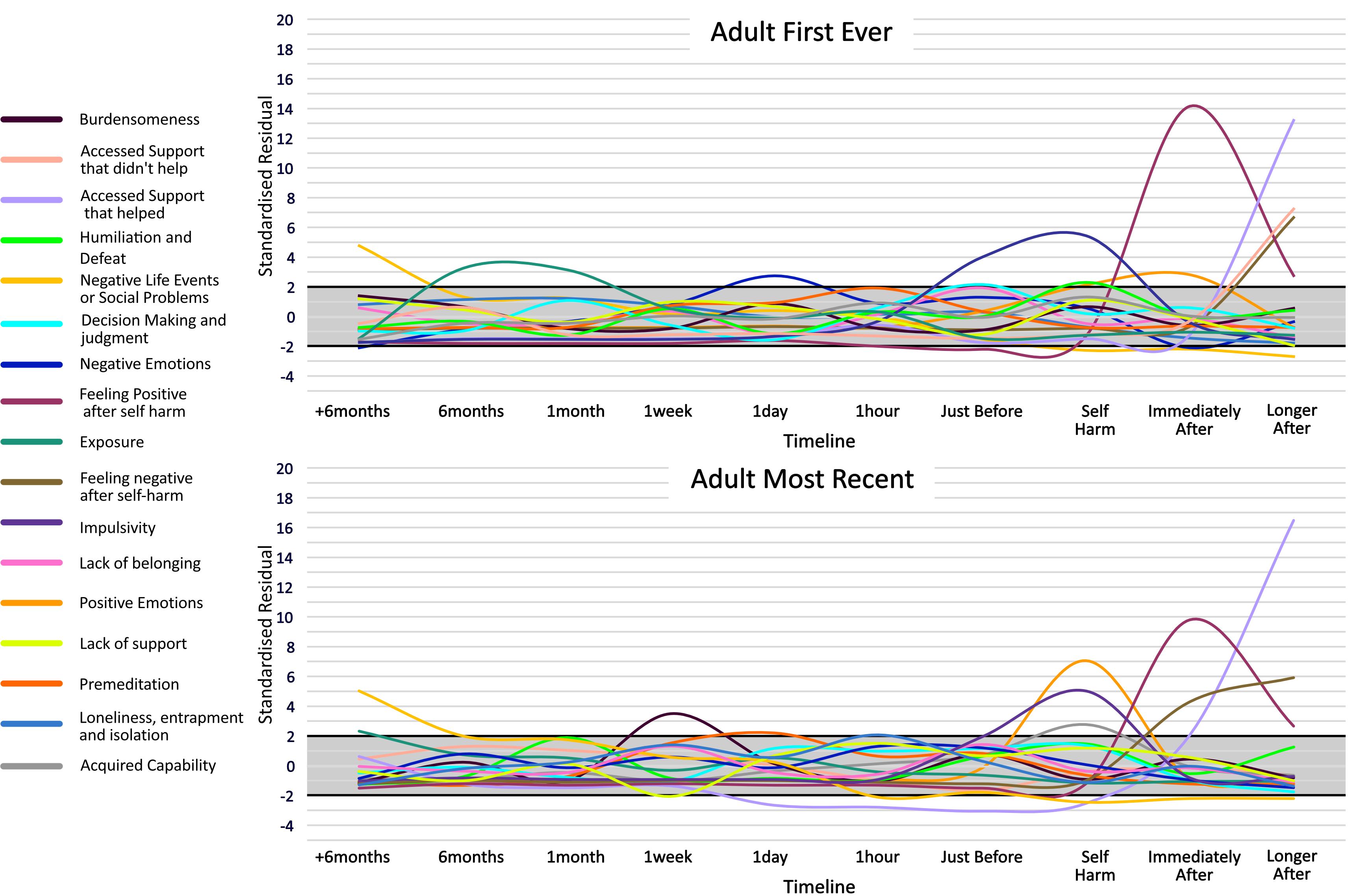


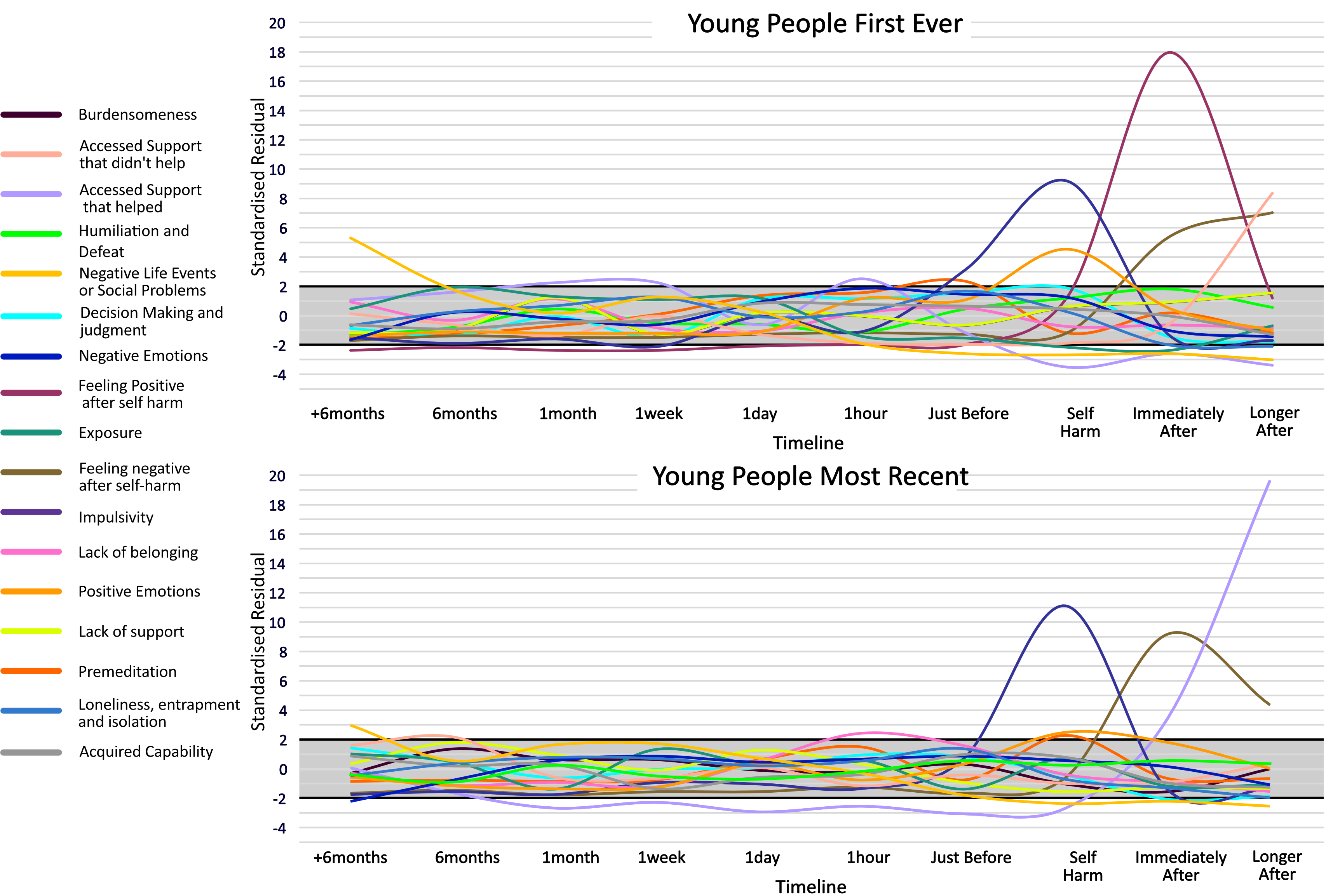

Supplement: Supplementary file 6 [file Table_6.DOCX]
