# Supplementary material for: A comparison of temporal pathways to self-harm in young people compared to adults: A pilot test of the Card Sort Task for Self-harm online using Indicator Wave Analysis
Source: Front Psychiatry. 2023 Jan 12;13:938003. doi: 10.3389/fpsyt.2022.938003 (PMC9878399; doi:10.3389/fpsyt.2022.938003)

S7 Indicator Wave graphs comparing the SRs for each group across the timeline, for each of the categories. Standardised residuals +/- 2 indicating higher or lower frequency than would be expected by chance.
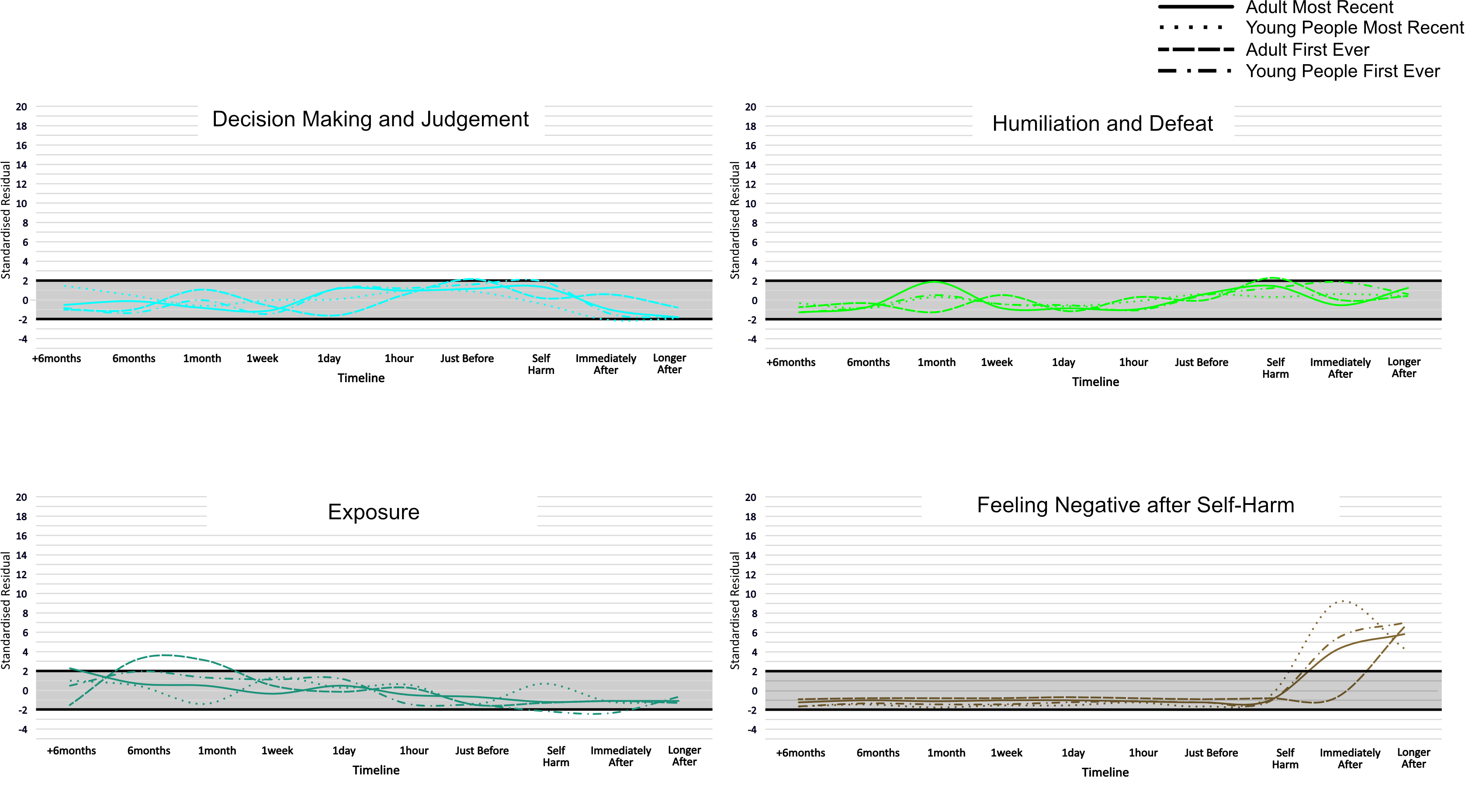


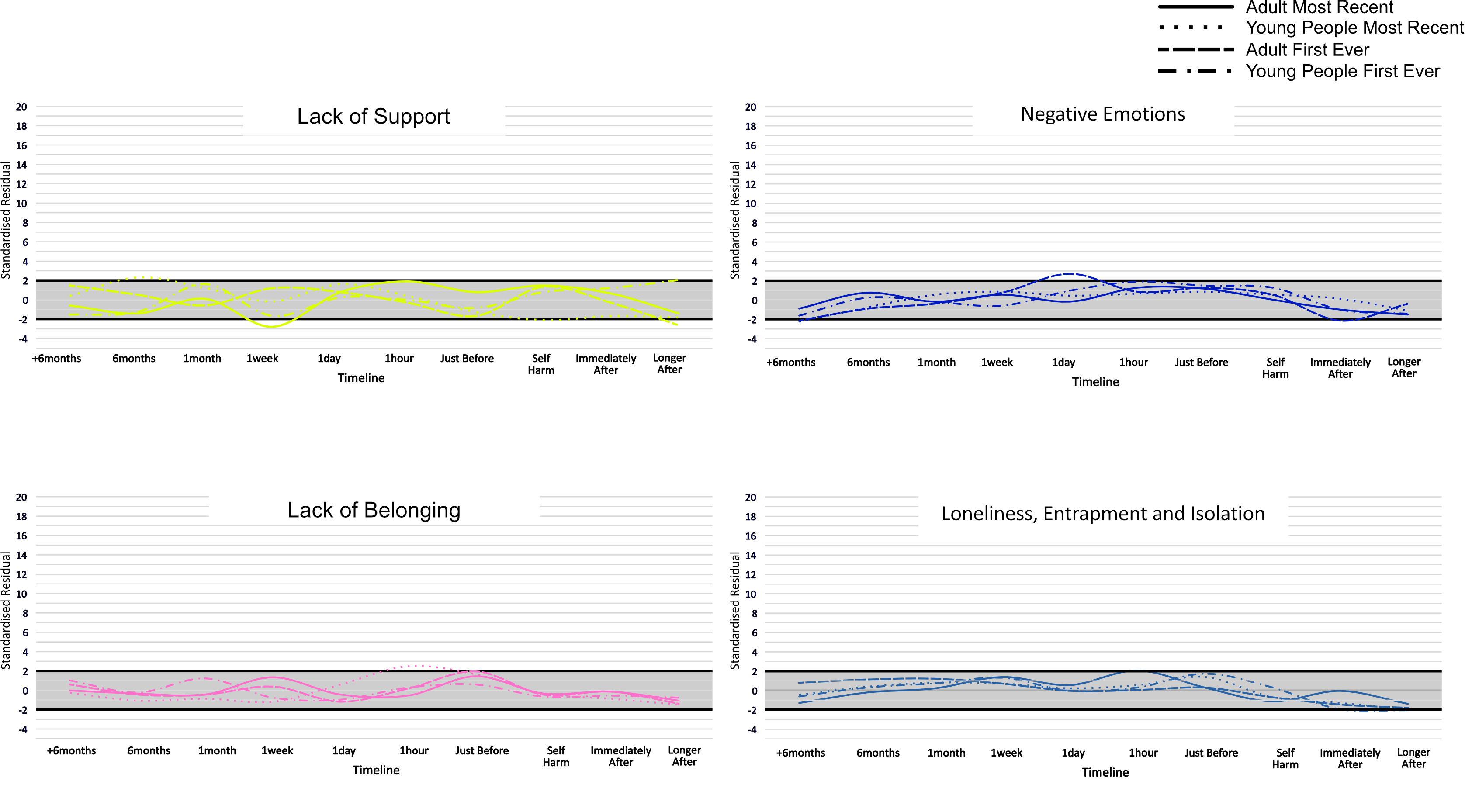

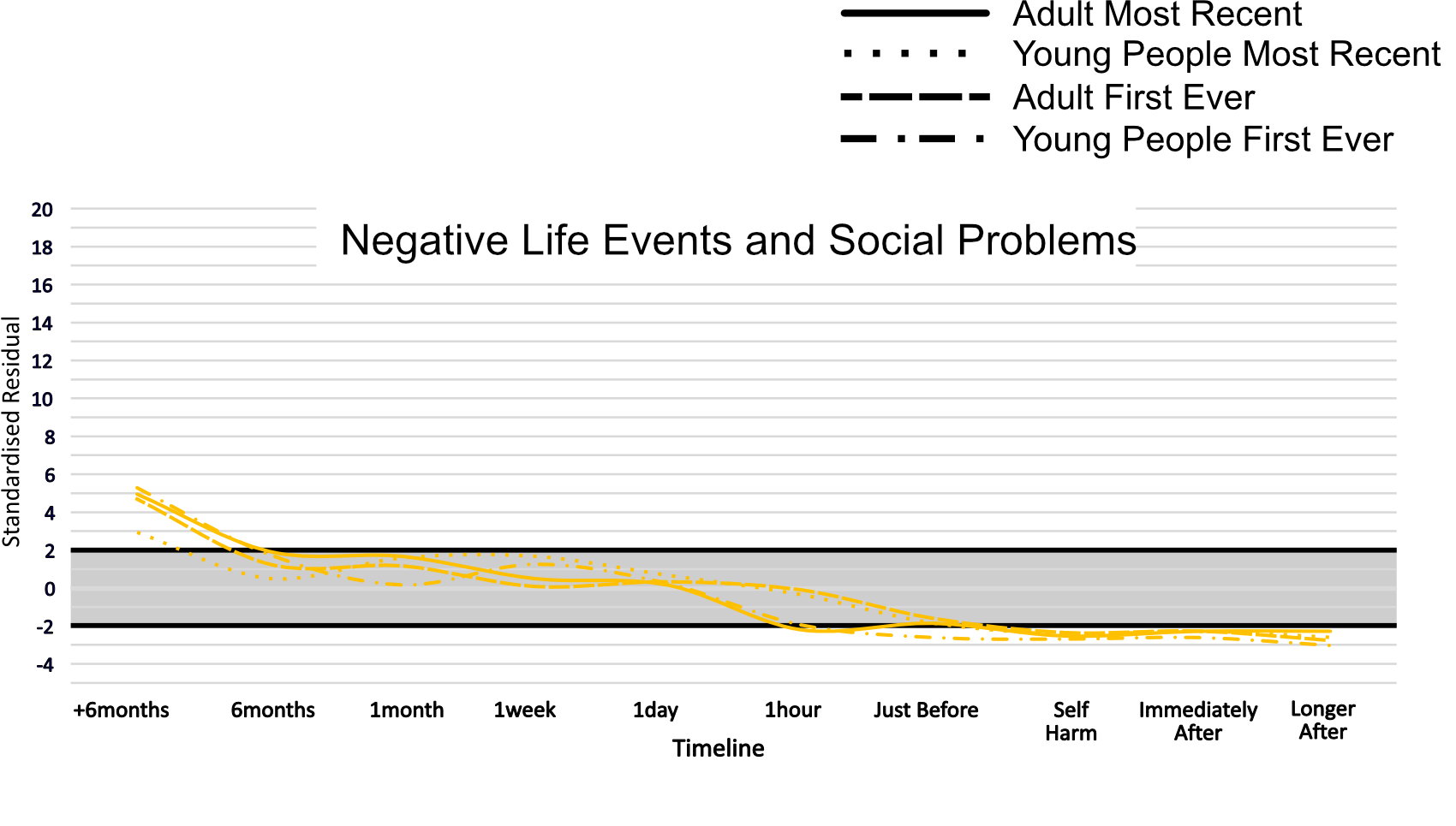

Supplement: Supplementary file 7 [file Table_7.DOCX]
